# Fracture of the Tibial Baseplate 16 Years after Miller-Galante II Total Knee Arthroplasty

**DOI:** 10.1155/2017/4080816

**Published:** 2017-05-16

**Authors:** Kazuaki Mineta, Masahiko Okada, Soshi Matsumoto, Daisuke Hamada, Tomohiro Goto, Koichi Sairyo

**Affiliations:** ^1^Department of Orthopedics, University of Tokushima, Tokushima, Japan; ^2^Department of Orthopedics, Tokushima Kensei Hospital, Tokushima, Japan; ^3^Department of Orthopedics, Kochi Seikyo Hospital, Tokushima, Japan

## Abstract

We report a rare case of tibial baseplate fracture of Miller-Galante II (MG II) prosthesis. We examine the factors that may cause such late fracture and review the literature on radiographic analysis and retrieval studies. A 76-year-old woman, who had undergone bilateral MG II total knee arthroplasty due to rheumatoid arthritis 16 years earlier, presented to our department with a 3-month history of left knee pain. Plain radiographs revealed severe distortion of the medial tibial component. During revision knee arthroplasty, we observed severe metallosis in the knee joint, polyethylene insert deformation, and posteromedial coronal baseplate fracture. After removal of the fractured tray, a bone deficit due to osteolysis was noted. The revision prosthesis (LCCK, Zimmer-Biomet) was implanted uneventfully. Four months after revision surgery, the patient was ambulating and had no complications. The implants on the right side had survived without complications for 17 years. We speculate that the primary causative factor of the fatigue fracture of the base plate due to loss of bony support most likely secondary to osteolysis was varus malalignment at primary implantation. This case highlights the importance of paying close attention to the correct alignment of each component at primary implantation.

## 1. Introduction

Total knee arthroplasty has constantly evolved since its introduction in 1968. Typically, a metal tibial baseplate is introduced beneath the polyethylene insert to improve the distribution of the forces between the implant and the bone and lower the incidence of implant failure attributable to loosening, subsidence, and polyethylene deformation [1]. However, several long-term complications of these procedures are well recognized, including polyethylene wear, osteolysis, metal debris synovitis, and fracture of the metal components [2, 3]. Of these, metal component fracture is a devastating complication that requires a highly complex revision arthroplasty procedure [4]. Fracture of the tibial baseplate is a relatively rare complication compared with fracture of the femoral component and Miller-Galante II (MG II) baseplate fracture is especially rare [5].

We report here a case of contralateral tibial baseplate fracture in a female patient who had undergone bilateral MG II total knee arthroplasty for bilateral rheumatoid arthritis of the knee 16 years earlier. We examine the factors that may cause such a late fracture on only one side and discuss radiographic analysis and retrieval studies reported in the literature.

## 2. Case Report

**Figure 1:**
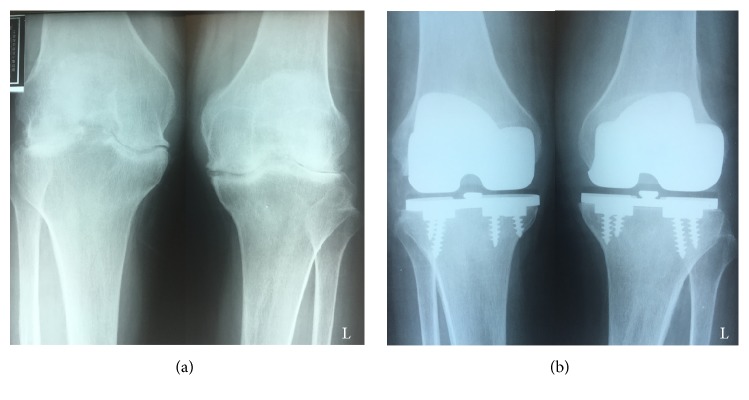
Plain radiographs of the bilateral knee of the patient with rheumatoid arthritis before and after Miller-Galante II Total Knee Arthroplasty performed 16 years earlier. (a) Preoperative plain radiograph shows bilateral rheumatoid arthritis of the knee categorized as Larsen grade 4. (b) Postoperative plain radiograph shows a femorotibial angle (FTA) of 174° on the right side and 183° on the left side. The left side shows varus malalignment.

Our patient was a 60-year-old woman who had undergone bilateral MG II (Zimmer) total knee arthroplasty for rheumatoid arthritis of the knee (Larsen grade IV) in our department 16 years earlier (Figure 1). She had no other relevant medical history. Body weight was 45.8 kg, height 158 cm, and body mass index 18.3 kg/m^2^. Weight and height were approximately the same as when the primary surgery was performed. After the initial procedure, she had been followed up every 3 months for 16 years and follow-up plain radiographs were regularly checked once a year. Nine years after the initial arthroplasty, she underwent surgery and chemotherapy for endometrial cancer; this treatment was successful and she experienced no recurrence or metastasis.

**Figure 2:**
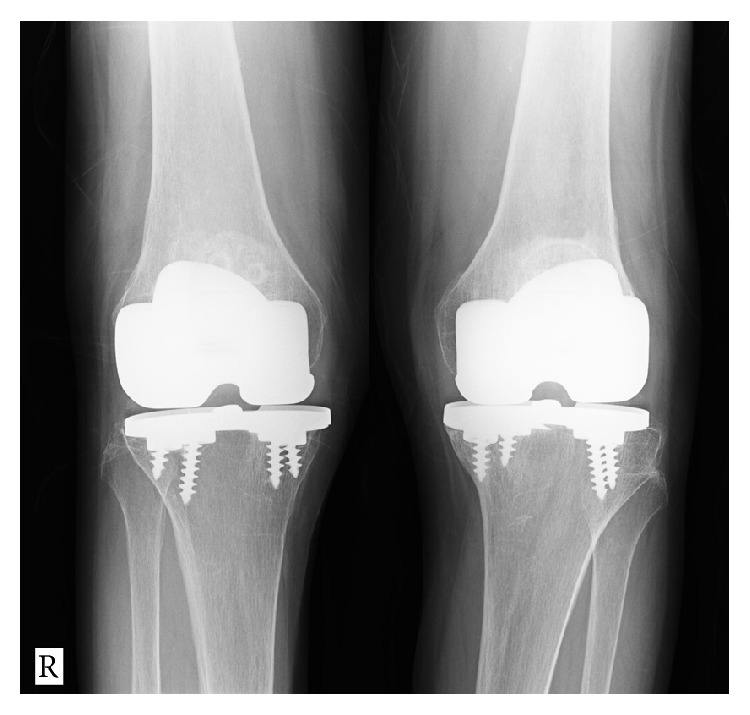
Follow-up plain radiograph obtained 15 years after surgery shows no change in the alignment of the right side and obvious deterioration of the varus malalignment (FTA of 190°) of the left side.

**Figure 3:**
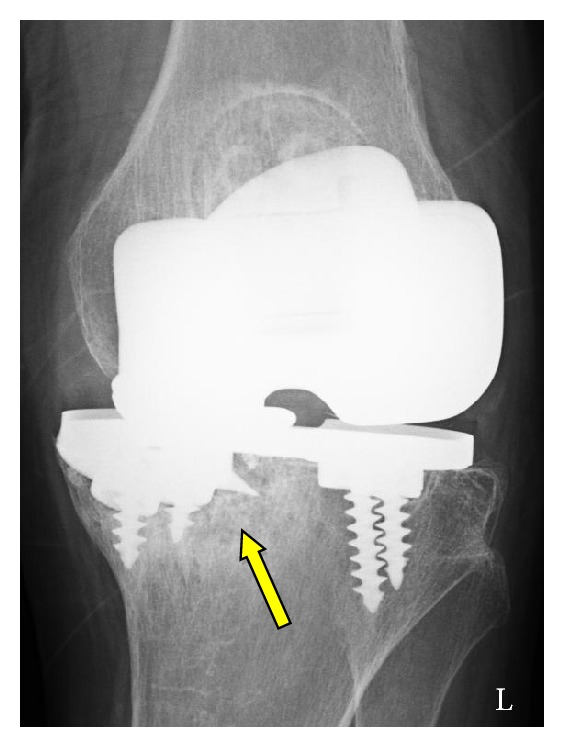
Follow-up plain radiograph obtained 16 years after the surgery shows disappearance of the medial joint space, subsidence of the broken baseplate, and osteolysis under the medial compartment (arrow).

A follow-up plain radiograph taken 15 years after arthroplasty revealed a deterioration of the varus malalignment of the tibia (Figure 2). However, the patient had no complaints about the left knee at this point, and we considered the problem was related to polyethylene wear. However, 16 years after arthroplasty, she presented to our department with a 3-month history of severe pain in the left knee. Clinically, she walked with a limp and a varus thrust. Examination of the left knee indicated joint effusion and tenderness along the medial joint line with localized warmth. There was a well-healed anterior skin incision over the left knee with no evidence of skin infection. Active and passive motions of the knee caused pain in the medial part of the knee. Plain radiographs taken at this time showed further distortion of the tibial component of the left knee with obvious fracture of the tibial baseplate and osteolysis under the broken component (Figure 3). She had not consulted us about the problem immediately because she had been undergoing conservative treatment for Graves's disease for 3 months. At the time of presentation, rheumatoid arthritis and Graves' disease were well controlled and all other laboratory values were in the normal range.

**Figure 4:**
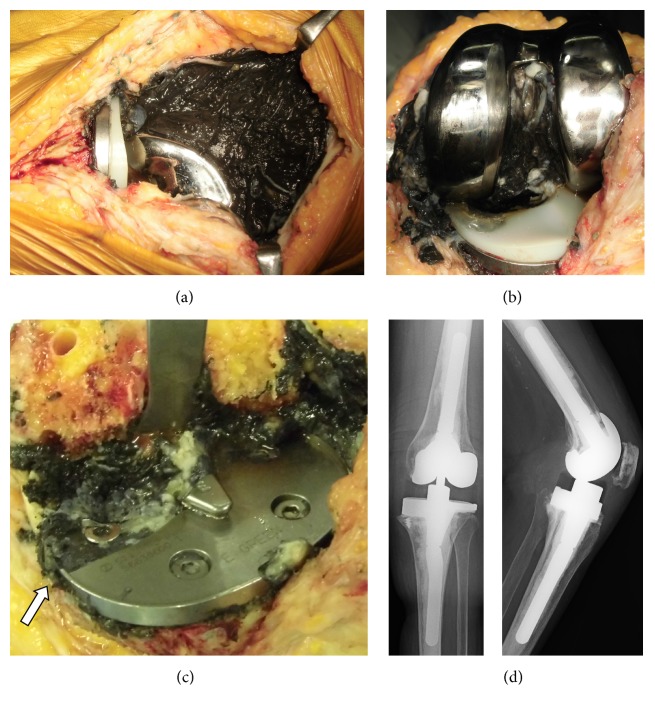
Gross appearance at revision surgery. (a) Severe metallosis is observed within the knee joint. (b) Polyethylene insert is heavily worn and broken at the posteromedial portion. (c) Coronal baseplate is fractured from the posteromedial corner to the posterior cruciate ligament recess (arrow). Subsidence of the posterior broken fragment of the baseplate is apparent.

**Figure 5:**
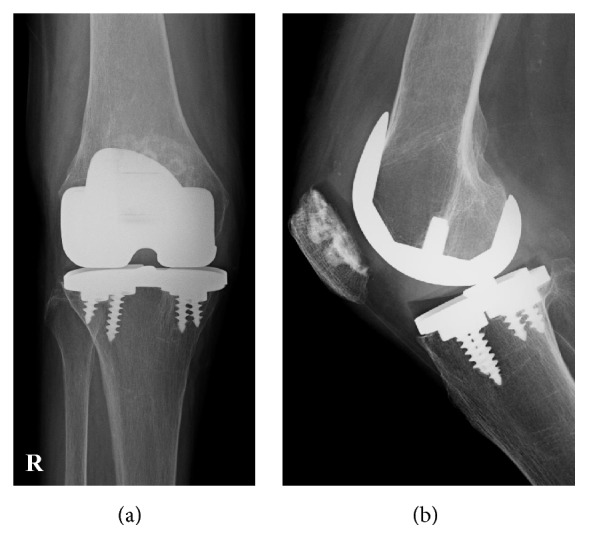
Follow-up plain radiograph obtained 17 years after initial surgery of the right-side implants shows normal alignment is maintained, with no evidence of stress shielding or loosening.

The patient underwent revision knee arthroplasty on the left side via the previous skin incision. Perioperatively, we observed severe metallosis within the knee joint (Figure 4(a)). The polyethylene insert was obviously worn and broken at the posteromedial site (Figure 4(b)). After the broken polyethylene insert was removed, a coronal fracture was found at the posteromedial portion of the tibial component and there was subsidence of the posterior metal tray fragment (Figure 4(c)). When the four screws were removed, we noted a cutting fracture of the screw inserted in the screw hole of the fractured tray. The remaining part of the screw could not be observed and we decided not to remove the remaining small screw fragment because it was embedded in bone and was unlikely to cause clinical complications. On extraction of the broken tibial metal tray, the medial aspect of the tibia showed cavitation of the cancellous bone under the medial weight-bearing surface. All metal and debris were removed and the revision prosthesis (LCCK, Zimmer-Biomet) was implanted. Medial augmentation was required to correct the bony deficiency (Figure 4(d)). There were no postoperative complications, and the patient was ambulating with full weight-bearing on postoperative day 2. Significant pain relief and functional improvement was achieved based on early follow-up examinations (4 months). Remarkably, the MG II prosthesis on the right side was properly aligned, surviving for 17 years after primary surgery (Figure 5).

**Figure 6:**
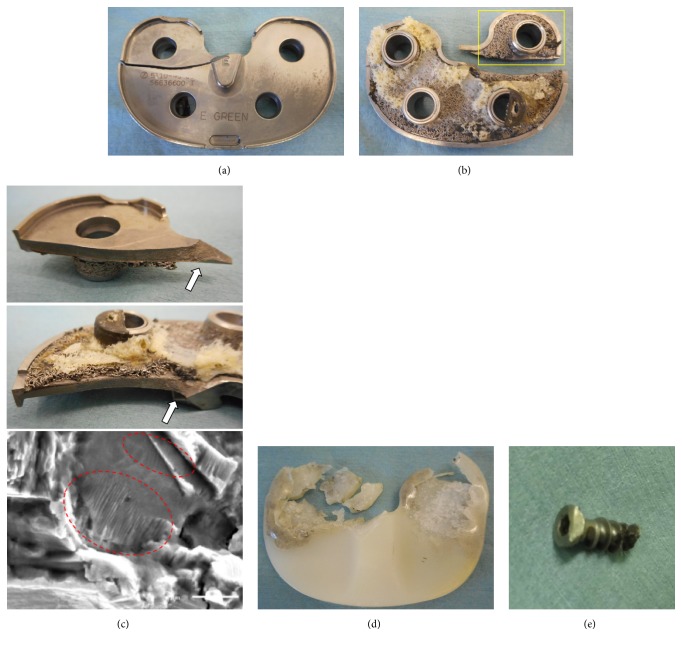
(a) Gross observation of the retrieved broken tibial baseplate reveals that the fracture line extended in a coronal direction from the medial corner to the posterior cruciate ligament (PCL) recess. (b) Gross observation of the undersurface of the tibial baseplate shows uneven bone attachment surrounding the medial fracture line but even bone attachment at the posterolateral portion. (c) Surface of the break is complex rather than with regular fracture waves. A focal stair-step pattern can be observed grossly at the medial corner of the PCL recess (arrow), indicating a fatigue fracture. Scanning electron micrography shows fatigue striations (circle). (d) Retrieved polyethylene insert has severely worn medial sites that correspond to the posteromedial breakage area of the tibial baseplate. Polyethylene insert is also severely worn at the posterolateral area. (e) Retrieved portion of the fractured screw.

Gross observation of the retrieved broken tibial baseplate revealed that the fracture line extended in a coronal direction from the medial corner to the posterior cruciate ligament (PCL) recess (Figure 6(a)). There was no evidence of bone ingrowth at the undersurface of the posterior fragment of the tibial tray, although bone ingrowth was noted under the tray anterior to the fracture line (Figure 6(b)). The broken surface was complex, instead of showing regular fracture waves. A focal stair-step pattern could be observed on gross examination at the medial corner of the PCL recess, and scanning electron micrography showed fatigue striations at this point (Figure 6(c)). This observation supported the possible development of a fracture line that had started at the medial corner of the PCL recess [6]. The retrieved polyethylene insert revealed that the heavily worn medial site corresponded to the posteromedial breakage area of the tibial baseplate (Figure 6(d)). The retrieved portion of the fractured screw demonstrated the severe load that had been exerted on the posterior fractured baseplate (Figure 6(e)).

## 3. Discussion

Total knee arthroplasty is associated with high survival rates in long-term follow-up studies [7–10], and the procedure has been constantly evolving. However, revision arthroplasty associated with knee arthroplasty failure has become a serious clinical problem. Polyethylene wear, osteolysis, and fatigue of the materials are the main problems that lead to implant failure [5, 11–13]. Fracture of the metallic tibial baseplate is a rare complication. Failure of the metallic tibial component is even more rare and has been reported to occur in about 1-2% of case at 10 years of follow-up [14]. Chatterji et al. [5] reported only 74 cases of tibial baseplate fracture, including 25 cases in their series, until 2005. We identified another 7 cases in the literature [6, 15–18]. Among these cases, breakage of the MG II tibial tray has been reported in only one other case [5]. Our case is likely to be the second such case among those reported to date.

Important factors implicated in tibial baseplate fractures are considered to include the design of the prostheses, surgical factors, poor tibial bone stock, obesity, and high activity [5, 6, 11, 12, 15–22]. Chatterji et al. [5], Abernethy et al. [19], and Flivik et al. [20] have indicated that manufacturing errors and inherent deficiency of the implant itself are also relevant factors, prompting well-designed implants to be manufactured. A long-term follow-up study of MG II showed an excellent survival rate over 10 years [23].

Ho et al. [6] have emphasized that the malalignment of implants can contribute to early tibial tray fractures even when a well-designed implant is used. Varus malalignment of implants exacerbates the compression load exerted on the medial portion during weight-bearing and on the posteromedial portion during flexion [24, 25]. In addition, external rotation of the tibial component relative to the femoral component may allow disproportionate load-bearing on the posteromedial corner, predisposing to a posterior coronal-type fracture [25]. On the other hand, there are also reports of fractures in prostheses with good alignment [5, 19]. Johnson et al. [26] and Harrington [27] have shown that, during normal gait, despite an apparently neutral or even valgus anatomical axis, the center of loading shifts to the medial compartment for most of the stance phase, which is the period of maximum load transmission. This may explain why fracture of the baseplate occurs despite apparently normal alignment. In our case, the patient was not obese and her activity level was comparatively restricted due to rheumatoid arthritis. Chatterji et al. [5] and Abernethy et al. [19] reported that baseplate fractures occurred more frequently in men than in women: our patient was female. Varus malalignment during the primary knee replacement was the main factor that contributed to the failure of the tibial tray in our patient. Even with poor alignment, low body weight and low activity levels and age might contribute to early breakage of such implants.

Gross observation of the knee during revision surgery and of the retrieved implants indicated another factor predisposing to the posteromedial coronal-type fracture observed in our case. On the undersurface of tibial baseplate, the fracture line passed through a region of uneven bone attachment. After removal of the fracture tray, we noted bone loss due to osteolysis, consistent with the radiographic appearance. In our case, the tibial baseplate was fixed inadequately due to osteolysis, resulting in cyclic cantilever bending that concentrated stresses at the junction of the supported and unsupported regions. This is a typical failure mechanism reported for tibial tray fractures [5, 15, 18, 19, 21, 22]. Cankaya et al. [18] reported polyethylene wear and osteolysis leading to tibial component fracture. We speculate that the malalignment of implants causes polyethylene wear and osteolysis, resulting in a bone deficit predisposing to this tibial component fracture. A review of 74 cases demonstrated 100% correlation between the site of the fractured tibial baseplate and the region of bone loss [5]. Gross observation of the surface of the fractured baseplate revealed a focal stair-step pattern at the medial corner of the PCL recess, indicating a fatigue fracture; this site was therefore considered to be the starting point of the fracture [15].

In our case, the implants on the right side were properly aligned and survived with no complaints for 17 years. We recognize that greater attention should have been paid to proper alignment and good ligament balancing on the left side, similar to that on the right side. Despite the use of modern, well-designed implants, the importance of regular follow-up examinations is clear from the present case.

## 4. Conclusion

Malposition of implants in total knee arthroplasty can cause devastating complications despite the use of a well-designed prosthesis. Our experience emphasizes the importance of proper alignment at the time of initial implantation and regular long-term follow-up.
